# A call for an open, informed study of all aspects of consciousness

**DOI:** 10.3389/fnhum.2014.00017

**Published:** 2014-01-27

**Authors:** Etzel Cardeña

**Affiliations:** Department of Psychology, Lund UniversityLund, Sweden

**Keywords:** consciousness, scientific method, parapsychology, PSI, psychical research

Science thrives when there is an open, informed discussion of all evidence, and recognition that scientific knowledge is provisional and subject to revision. This attitude is in stark contrast with reaching conclusions based solely on a previous set of beliefs or on the assertions of authority figures. Indeed, the search for knowledge wherever it may lead inspired a group of notable scientists and philosophers to found in 1882 the Society for Psychical Research in London. Its purpose was “to investigate that large body of debatable phenomena… without prejudice or prepossession of any kind, and in the same spirit of exact and unimpassioned inquiry which has enabled Science to solve so many problems.” Some of the areas in consciousness they investigated such as psychological dissociation, hypnosis, and preconscious cognition are now well integrated into mainstream science. That has not been the case with research on phenomena such as purported telepathy or precognition, which some scientists (a clear minority according to the surveys conducted http://en.wikademia.org/Surveys_of_academic_opinion_regarding_parapsychology) dis-miss *a priori* as pseudoscience or illegitimate. Contrary to the negative impression given by some critics, we would like to stress the following:

1. Research on parapsychological phenomena (psi) is being carried out in various accredited universities and research centers throughout the world by academics in different disciplines trained in the scientific method (e.g., circa 80 Ph.D.s have been awarded in psi-related topics in the UK in recent years). This research has continued for over a century despite the taboo against investigating the topic, almost complete lack of funding, and professional and personal attacks (Cardeña, 201). The Parapsychological Association has been an affiliate of the AAAS since 1969, and more than 20 Nobel prizewinners and many other eminent scientists have supported the study of psi or even conducted research themselves (Cardeña, 2013).
2. Despite a negative attitude by some editors and reviewers, results supporting the validity of psi phenomena continue to be published in peer-reviewed, academic journals in relevant fields, from psychology to neuroscience to physics e.g., (Storm et al., 2010; Bem, 2011; Hameroff, 2012; Radin et al., 2012).
3. Increased experimental controls have not eliminated or even decreased significant support for the existence of psi phenomena, as suggested by various recent meta-analyses (Sherwood and Roe, 2003; Schmidt et al., 2004; Bösch et al., 2006; Radin et al., 2006; Storm et al., 2010, 2012, 2013; Tressoldi, 2011; Mossbridge et al., 2012; Schmidt, 2012).
4. These meta-analyses and other studies (Blackmore, 1980)suggest that data supportive of psi phenomena cannot reasonably be accounted for by chance or by a “file drawer” effect. Indeed, contrary to most disciplines, parapsychology journals have for decades encouraged publication of null results and of papers critical of a psi explanation (Wiseman et al., 1996; Schönwetter et al., 2011). A psi trial registry has been established to improve research practice http://www.koestler-parapsychology.psy.ed.ac.uk/TrialRegistryDetails.html.
5. The effect sizes reported in most meta-analyses are relatively small and the phenomena cannot be produced on demand, but this also characterizes various phenomena found in other disciplines that focus on complex human behavior and performance such as psychology and medicine (Utts, 1991; Richard and Bond, 2003).
6. Although more conclusive explanations for psi phenomena await further theoretical and research developments, they do not *prima facie* violate known laws of nature given modern theories in physics that transcend classical restrictions of time and space, combined with growing evidence for quantum effects in biological systems (Sheehan, 2011; Lambert et al., 2013).

With respect to the proposal that “exceptional claims require exceptional evidence,” the original intention of the phrase is typically misunderstood (Truzzi, 1978). Even in its inaccurate interpretation what counts as an “exceptional claim” is far from clear. For instance, many phenomena now accepted in science such as the existence of meteorites, the germ theory of disease, or, more recently, adult neurogenesis, were originally considered so exceptional that evidence for their existence was ignored or dismissed by contemporaneous scientists. It is also far from clear what would count as “exceptional evidence” or who would set that threshold. Dismissing empirical observations *a priori*, based solely on biases or theoretical assumptions, underlies a distrust of the ability of the scientific process to discuss and evaluate evidence on its own merits. The undersigned differ in the extent to which we are convinced that the case for psi phenomena has already been made, but not in our view of science as a non-dogmatic, open, critical but respectful process that requires thorough consideration of all evidence as well as skepticism toward both the assumptions we already hold and those that challenge them.

Daryl Bem, Professor Emeritus of Psychology, Cornell University, USA

Etzel Cardeña, Thorsen Professor of Psychology, Lund University, Sweden

Bernard Carr, Professor in Mathematics and Astronomy, University of London, UK

C. Robert Cloninger, Renard Professor of Psychiatry, Genetics, and Psychology, Washington University in St. Louis, USA

Robert G. Jahn, Past Dean of Engineering, Princeton University, USA

Brian Josephson, Emeritus Professor of Physics, University of Cambridge, UK (Nobel prizewinner in physics, 1973)

Menas C. Kafatos, Fletcher Jones Endowed Professor of Computational Physics, Chapman University, USA

Irving Kirsch, Professor of Psychology, University of Plymouth, Lecturer in Medicine, Harvard Medical School, USA, UK

Mark Leary, Professor of Psychology and Neuroscience, Duke University, USA

Dean Radin, Chief Scientist, Institute of Noetic Sciences, Adjunct Faculty in Psychology, Sonoma State University, USA

Robert Rosenthal, Distinguished Professor, University of California, Riverside, Edgar Pierce Professor Emeritus, Harvard University, USA

Lothar Schäfer, Distinguished Professor Emeritus of Physical Chemistry, University of Arkansas, USA

Raymond Tallis, Emeritus Professor of Geriatric Medicine, University of Manchester, UK

Charles T. Tart, Professor in Psychology Emeritus, University of California, Davis, USA

Simon Thorpe, Director of Research CNRS (Brain and Cognition), University of Toulouse, France

Patrizio Tressoldi, Researcher in Psychology, Università degli Studi di Padova, Italy

Jessica Utts, Professor and Chair of Statistics, University of California, Irvine, USA

Max Velmans, Professor Emeritus in Psychology, Goldsmiths, University of London, UK

Caroline Watt, Senior Lecturer in Psychology, Edinburgh University, UK

Phil Zimbardo, Professor in Psychology Emeritus, Stanford University, USA

And…

P. Baseilhac, Researcher in Theoretical Physics, University of Tours, France

Eberhard Bauer, Dept. Head, Institute of Border Areas of Psychology and Mental Hygiene, Freiburg, Germany

Julie Beischel, Adjunct Faculty in Psychology and Integrated Inquity, Saybrook University, USA

Hans Bengtsson, Professor of Psychology, Lund University, Sweden

Michael Bloch, Associate Professor of Psychology, University of San Francisco, USA

Stephen Braude, Professor of Philosophy Emeritus, University of Maryland Baltimore County, USA

Richard Broughton, Senior Lecturer, School of Social Sciences, University of Northampton, UK

Antonio Capafons, Professor of Psychology, University of Valencia, Spain

James C. Carpenter, Adjunct Professor of Psychiatry, University of North Carolina, Chapel Hill, USA

Allan Leslie Combs, Doshi Professor of Consciousness Studies, California Institute of Integral Studies, USA

Deborah Delanoy, Emeritus Professor of Psychology, University of Northampton, UK

Arnaud Delorme, Professor of Neuroscience, Paul Sabatier University, France

Vilfredo De Pascalis, Professor of General Psychology, “La Sapienza” University of Rome, Italy

Kurt Dressler, Professor in Molecular Spectroscopy Emeritus, Eidg. Techn. Hochschule Zürich, Switzerland

Hoyt Edge, Hugh H. and Jeannette G. McKean Professor of Philosophy, Rollins College, USA

Suitbert Ertel, Emeritus Professor of Psychology, University of Göttingen, Germany

Franco Fabbro, Professor in Child Neuropsychiatry, University of Udine, Italy

Enrico Facco, Professor of Anesthesia and Intensive Care, University of Padua, Italy

Wolfgang Fach, Researcher, Institute of Border Areas of Psychology and Mental Hygiene, Freiburg, Germany

Harris L. Friedman, Former Research Professor of Psychology, University of Florida, USA

Alan Gauld, Former Reader in Psychology, University of Nottingham, UK

Antoon Geels, Professor in the Psychology of Religion Emeritus, Lund University, Sweden

Bruce Greyson, Carlson Professor of Psychiatry and Neurobehavioral Sciences, University of Virginia, Charlottesville, USA

Erlendur Haraldsson, Professor Emeritus of Psychology, University of Iceland, Iceland

Richard Conn Henry, Academy Professor (Physics and Astronomy), The Johns Hopkins University, USA

David J. Hufford, University Professor Emeritus, Penn State College of Medicine, USA

Oscar Iborra, Researcher, Department of Experimental Psychology, Granada University, Spain

Harvey Irwin, former Associate Professor, University of New England, Australia

Graham Jamieson, Lecturer in Human Neuropsychology, University of New England, Australia

Erick Janssen, Adjunct Professor, Department of Psychology, Indiana University, USA

Per Johnsson, Head, Department of Psychology, Lund University, Sweden

Edward F. Kelly, Research Professor in the Department of Psychiatry and Neurobehavioral Sciences, University of Virginia, Charlottesville, USA

Emily Williams Kelly, Research Assistant Professor in the Department of Psychiatry and Neurobehavioral Sciences, University of Virginia, Charlottesville, USA

Hideyuki Kokubo, Researcher, Institute for Informatics of Consciousness, Meiji University, Japan

Jeffrey J. Kripal, J. Newton Rayzor Professor of Religious Studies, Rice University, USA

Stanley Krippner, Professor of Psychology and Integrated Inquiry, Saybrook University, USA

David Luke, Senior Lecturer, Department of Psychology and Counselling, University of Greenwich, UK

Fatima Regina Machado, Researcher, Universidade de São Paulo, Brasil

Markus Maier, Professor in Psychology, University of Munich, Germany

Gerhard Mayer, Researcher, Institute of Border Areas of Psychology and Mental Hygiene, Freiburg, Germany

Antonia Mills, Professor First Nations Studies, University of Northern British Columbia, Canada

Garret Moddel, Professor in Electrical, Computer, & Energy Engineering, University of Colorado, Boulder, USA

Alexander Moreira-Almeida, Professor of Psychiatry, Universidade Federal de Juiz de Fora, Brasil

Andrew Moskowitz, Professor in Psychology and Behavioral Sciences, Aarhus University, Denmark

Julia Mossbridge, Fellow in Psychology, Northwestern University, USA

Judi Neal, Professor Emeritus of Management, University of New Haven, USA

Roger Nelson, Retired Research Staff, Princeton University, USA

Fotini Pallikari, Professor of Physics, University of Athens, Greece

Alejandro Parra, Researcher in Psychology, Universidad Abierta Interamericana, Argentina

José Miguel Pérez Navarro, Lecturer in Education, International University of La Rioja, Spain

Gerald H. Pollack, Professor in Bioengineering. University of Washington, Seattle, USA

John Poynton, Professor Emeritus in Biology, University of KwaZulu-Natal, South Africa

David Presti, Senior Lecturer, Neurobiology and Cognitive Science, University of California, Berkeley, USA

Thomas Rabeyron, Lecturer in Clinical Psychology, Nantes University, France

Inmaculada Ramos Lerate, Researcher in Physics, Alba Synchrotron Light Source, Barcelona, Spain.

Chris Roe, Professor of Psychology, University of Northampton, UK

Stefan Schmidt, Professor, Europa Universität Viadrina, Germany

Gary E. Schwartz, Professor of Psychology, Medicine, Neurology, Psychiatry, and Surgery, University of Arizona, USA

Daniel P. Sheehan, Professor of Physics, University of San Diego, USA

Simon Sherwood, Senior Lecturer in Psychology, University of Greenwich, UK

Christine Simmonds-Moore, Assistant Professor of Psychology, University of West Georgia, USA

Mário Simões, Professor in Psychiatry. University of Lisbon, Portugal

Huston Smith, Prof. of Philosophy Emeritus, Syracuse University, USA

Jerry Solfvin, Associate Professor in Indic Studies, University of Massachusetts, Dartmouth, USA

Lance Storm, Visiting Research Fellow, University of Adelaide, Australia

Jeffrey Allan Sugar, Assistant Professor of Clinical Psychiatry, University of Southern California, Los Angeles, USA

Neil Theise, Professor of Pathology and Medicine, The Icahn School of Medicine at Mount Sinai, USA

Jim Tucker, Bonner-Lowry Associate Professor of Psychiatry and Neurobehavioral Sciences, University of Virginia, USA

Yulia Ustinova, Associate Professor in History, Ben-Gurion University of the Negev, Israel

Walter von Lucadou, Senior Lecturer at the Furtwangen Technical University, Germany

Maurits van den Noort, Senior Researcher, Free University of Brussels, Belgium

David Vernon, Senior Lecturer in Psychology, Canterbury Christ Church University, UK

Harald Walach, Professor, Europa Universität Viadrina, Germany

Helmut Wautischer, Senior Lecturer in Philosophy, Sonoma State University, USA

Donald West, Emeritus Professor of Clinical Criminology, University of Cambridge, UK

N.C. Wickramasinghe, Professor in Astrobiology, Cardiff University, UK

Fred Alan Wolf, formerly Professor in physics at San Diego State University, the Universities of Paris, London, and the Hebrew University of Jerusalem

Robin Wooffitt, Professor of Sociology, University of York, UK

Wellington Zangari, Professor in Psychology, University of Sao Paulo, Brazil

Aldo Zucco, Professor, Dipartimento di Psicologia Generale, Università di Padova, Italy
